# Comparison of 2 Disability Measures, Behavioral Risk Factor Surveillance System, 2013

**DOI:** 10.5888/pcd13.160080

**Published:** 2016-08-11

**Authors:** Alissa C. Stevens, Elizabeth A. Courtney-Long, Catherine A. Okoro, Dianna D. Carroll

**Affiliations:** Author Affiliations: Elizabeth A. Courtney-Long, Catherine A. Okoro, Centers for Disease Control and Prevention, Atlanta, Georgia; Dianna D. Carroll, Centers for Disease Control and Prevention and Commissioned Corps, US Public Health Service, Atlanta, Georgia.

## Abstract

**Introduction:**

Beginning in 2013, in addition to the 2-item disability question set asked since 2001, Behavioral Risk Factor Surveillance System (BRFSS) began using 5 of the 6 items from the US Department of Health and Human Services–recommended disability question set. We assess and compare disability prevalence using the 2-question and 5-question sets and describe characteristics of respondents who identified as having a disability using each question set.

**Methods:**

We used data from the 2013 BRFSS to estimate the prevalence of disability for each question set and the 5 specific types of disability. Among respondents identified by each disability question set, we calculated the prevalence of selected demographic characteristics, health conditions, health behaviors, and health status.

**Results:**

With the 2-question set, 21.6% of adults had a disability and with the 5-question set, 22.7% of adults had disability. A total of 51.2% of adults who identified as having a disability with either the 2-question or 5-question set reported having disabilities with both sets. Adults with different disability types differed by demographic and health characteristics.

**Conclusion:**

The inclusion of the 5 new disability questions in BRFSS provides a level of detail that can help develop targeted interventions and programs and can guide the adaptation of existing health promotion programs to be more inclusive of adults who experience specific types of disabilities.

## Introduction

Adults with disabilities represent a significant proportion (approximately 20%–30%) of the US population (1–3), experience health disparities (4–6), and have higher health care utilization (7–9). To monitor the health of this population, measures identifying adults with disabilities are often included in public health surveillance systems. However, disability is a complex, multidimensional concept that is difficult to fully assess with few questions on a survey (2,3). As a result, several questions have been used in large-scale population-based surveys that identify different aspects of disability and therefore yield different estimates (2,3,10–12). These differences are partly due to inconsistent measurement of disability, but also to variation in survey methods (eg, mode of data collection, sampling frame and design).

In 2013, the Behavioral Risk Factor Surveillance System (BRFSS) included 2 question sets that each measure disability. The first, a 2-question measure included on the questionnaire since 2001, assesses general activity limitation and special equipment use (11). The second is a 5-question measure that assesses serious difficulty in vision, cognition, or ambulation and any difficulty in self-care or independent living and is a subset of a standard 6-question set recommended by the US Department of Health and Human Services (HHS) for inclusion in population-based health surveys (12). Although the 2013 questionnaire did not include the sixth HHS-recommended question that assesses serious difficulty hearing, this question was included on the questionnaire beginning in 2016.

The inclusion of these 2 question sets in BRFSS provides a unique opportunity to compare 2 measures of disability. Any differences noted will result from differences in how disability is measured rather than methodological considerations, as when comparing disability estimates across surveys (13–16). The purpose of this study was to compare the prevalence of disability measured by the 2 question sets and to describe demographic and health characteristics of respondents who identified as having a disability using each measure.

## Methods

We used data from 2013 BRFSS, an annual state-based telephone survey of the civilian, noninstitutionalized US population that collects information on demographics, health behaviors, and health conditions (17). All 50 states, the District of Columbia, Guam, and Puerto Rico collected data from both landline and cellular telephone respondents. Response rates for BRFSS are calculated as the proportion of respondents who completed the survey among all eligible and possibly eligible persons using standards set by the American Association of Public Opinion Research Response Rate Formula no. 4 (http://www.aapor.org/Content/NavigationMenu/ResourcesforResearchers/StandardDefinitions/StandardDefinitions2009new.pdf). The median survey response rate for 2013 for all states, territories, and the District of Columbia was 46.4%, ranging from 29.0% to 60.3% (18). Data were collected for 491,773 respondents, of whom 487,044 respondents reported a valid age of 18 years or older. We excluded respondents with missing, “refused”, or “don’t know” responses for any of the 7 disability questions (n = 20,298), resulting in an analytic sample of 466,746 respondents.

Respondents were categorized as having a disability according to the 2-question measure if they answered yes to either of the following questions: 1) “Are you limited in any way in any activities because of physical, mental, or emotional problems?” (an activity limitation); and 2) “Do you now have any health problem that requires you to use special equipment, such as a cane, a wheelchair, a special bed, or a special telephone?” (use of special equipment). Respondents who answered no to both questions were considered not to have a disability according to this measure.

Respondents were categorized as having a disability according to the 5-question measure if they answered yes to any of the following questions: 1) “Are you blind or do you have serious difficulty seeing, even when wearing glasses?” (a vision disability); 2) “Because of a physical, mental, or emotional condition, do you have serious difficulty concentrating, remembering, or making decisions?” (a cognitive disability); 3) “Do you have serious difficulty walking or climbing stairs?” (an ambulatory disability); 4) “Do you have difficulty dressing or bathing?” (a self-care disability); and 5) “Because of a physical, mental, or emotional condition, do you have difficulty doing errands alone, such as visiting a doctor’s office or shopping?” (an independent living disability). Respondents who answered no to all 5 questions were considered not to have a disability according to this measure.

Respondents could identify as having a disability with either or both the 2-question and 5-question sets; thus, these categories were not mutually exclusive. Therefore, we also calculated a variable with mutually exclusive disability groups: both 2-question and 5-question disability, 2-question disability only, and 5-question disability only. We did not combine all 7 questions into a single measure of disability, because the 2-question and 5-question sets are intended to represent distinct measures of disability. To analyze the types of disability identified by the 5-question set, we calculated a variable with mutually exclusive disability types: vision disability only, cognitive disability only, ambulatory disability only, self-care disability only, independent living disability only, and 2 or more types of disability.

We used SAS-callable SUDAAN version 11.0 (RTI International) to account for complex survey design. Analyses were weighted to account for survey noncoverage and nonresponse and to adjust for demographic characteristics such as age, sex, race/ethnicity, and telephone ownership. We estimated the overall prevalence of disability for each question set (ie, disability identified by the 2-question set or the 5-question set) as well as the prevalence of 5 specific disability types. Among respondents identified by each disability measure (2-question set, 5-question set, and 5 types of disability), we calculated the prevalence of selected demographic characteristics (age group, sex, race/ethnicity), health conditions (arthritis, heart disease, diabetes, obesity, depression, hypertension, or 2 or more of these conditions), health behaviors (current smoking [smoked at least 100 cigarettes in their lifetime and currently smoked either every day or some days], binge drinking in the past 30 days [consumed at least 5 drinks (men) or at least 4 drinks (women) on an occasion one or more times during the past 30 days], receipt of an influenza [flu] vaccine in the past year, engaging in sufficient aerobic physical activity [participated in ≥150 minutes of moderate-intensity equivalent aerobic activity per week]), and health status (fair or poor self-rated health, ≥14 poor physical health days in the past 30 days, ≥14 poor mental health days in the past 30 days).

## Results

More than 80% of the weighted adult sample was younger than 65 years, more than half were female, and nearly two-thirds were non-Hispanic white. Hypertension was the most commonly reported health condition (32.5%), followed by obesity (28.4%) and arthritis (25.1%). Overall, 18.1% (95% confidence interval [CI], 17.9%–18.4%) of adults were current smokers, 16.7% (95% CI, 16.4%–16.9%) reported binge drinking, 38.5% (95% CI, 38.2%–38.7%) reported getting a flu vaccine in the past year, and 49.8% (95% CI, 49.5%–50.1%) reported engaging in sufficient aerobic physical activity. Fair or poor self-rated health was reported by 17.8% (95% CI, 17.6%–18.0%) of adults, and 14 or more physically and mentally unhealthy days were reported by 12.0% (95% CI, 11.8%–12.2%) and 11.3% (95% CI, 11.1%–11.5%) of adults, respectively. Disability prevalence using the 2-question measure was 21.6% (95% CI, 21.4%–21.8%) and 22.7% (95% CI, 22.4%–22.9%) using the 5-question measure. Overall, having an activity limitation (19.7%) was the most frequently reported item on the 2-question measure, while ambulatory disability (13.8%) was the most commonly reported disability type of the 5-question measure (Table 1).

**Table 1:** Prevalence of Selected Demographic and Health Characteristics of the Overall Population and by Nonmutually Exclusive Disability Category^a^ Among Adults Aged 18 Years or Older, United States and Territories, 2013 Behavioral Risk Factor Surveillance System

Characteristic	Overall (N = 466,746)		2-Question Disability (N = 124,550)		5-Question Disability (N = 120,565)	
	n	Weighted % (95% CI)	n	Weighted % (95% CI)	n	Weighted% (95% CI)
**Age group, y**						
18–24	26,028	13.1 (12.9–13.4)	2,447	5.7 (5.3–6.0)	3,548	8.5 (8.1–8.9)
25–44	105,455	33.7 (33.4–34.0)	15,117	21.5 (21.0–22.0)	15,618	23.4 (22.8–23.9)
45–64	182,328	34.5 (34.2–34.8)	51,905	42.9 (42.3–43.5)	47,324	39.3 (38.7–39.9)
≥65	152,935	18.7 (18.5–18.9)	55,081	30.0 (29.5–30.4)	54,075	28.8 (28.4–29.3)
**Sex**						
Male	191,399	48.6 (48.3–48.9)	48,083	46.0 (45.4–46.6)	42,139	42.6 (42.0–43.2)
Female	275,347	51.4 (51.1–51.7)	76,467	54.0 (53.4–54.6)	78,426	57.4 (56.8–58.0)
**Race/ethnicity**						
White, non-Hispanic	359,826	65.1 (64.7–65.4)	97,476	70.2 (69.6–70.8)	88,308	62.5 (61.8–63.1)
Black, non-Hispanic	36,515	11.5 (11.3–11.7)	10,590	12.5 (12.1–12.9)	12,235	14.4 (14.0–14.9)
Hispanic	34,861	16.1 (15.8–16.4)	7,047	11.4 (11.0–11.9)	10,104	16.8 (16.3–17.4)
Other, non-Hispanic	28,671	7.4 (7.2–7.6)	7,317	5.9 (5.5–6.3)	7,847	6.3 (5.9–6.7)
**Health conditions**						
Arthritis	156,401	25.1 (24.8–25.3)	77,760	56.6 (56.0–57.2)	73,071	51.8 (51.2–52.4)
Heart disease	41,979	6.7 (6.5–6.8)	23,546	16.6 (16.2–17.0)	22,932	16.0 (15.6–16.5)
Diabetes	58,824	10.2 (10.1–10.4)	28,588	21.3 (20.8–21.8)	29,277	21.3 (20.8–21.8)
Obesity	129,395	28.4 (28.1–28.6)	47,761	40.3 (39.7–40.9)	46,722	40.2 (39.6–40.8)
Depression	91,090	17.8 (17.6–18.0)	46,211	39.2 (38.6–39.8)	47,275	40.9 (40.3–41.5)
Hypertension	188,350	32.5 (32.3–32.8)	72,313	52.7 (52.1–53.3)	71,046	51.5 (50.9–52.1)
≥2 Chronic conditions^b^	193,226	33.4 (33.1–33.7)	88,663	66.9 (66.3–67.5)	86,663	65.1 (64.5–65.7)
**Health behaviors**						
Current smoking^c^	74,635	18.1 (17.9–18.4)	26,072	25.3 (24.7–25.8)	27,667	27.3 (26.8–27.9)
Binge drinking^d^	58,013	16.7 (16.4–16.9)	10,083	11.6 (11.2–12.0)	9,878	12.4 (12.0–12.9)
Flu vaccine	203,034	38.5 (38.2–38.7)	62,186	46.5 (45.9–47.1)	57,377	43.5 (42.8–44.1)
Sufficient aerobic physical activity^e^	220,763	49.8 (49.5–50.1)	44,924	38.5 (37.9–39.1)	39,658	36.4 (35.8–37.0)
**Health status**						
Fair or poor self-rated health	88,122	17.8 (17.6–18.0)	58,017	47.1 (46.5–47.6)	59,295	48.0 (47.4–48.6)
≥14 Poor physical health days	61,706	12.0 (11.8–12.2)	45,688	38.5 (37.9–39.1)	44,143	36.4 (35.8–37.0)
≥14 Poor mental health days	47,363	11.3 (11.1–11.5)	27,229	26.6 (26.0–27.1)	29,420	29.4 (28.8–30.0)
**2-question disability^a^**	124,550	21.6 (21.4–21.8)	—	—	—	—
Activity limitation	112,049	19.7 (19.5–19.9)	—	—	—	—
Use of special equipment	52,988	8.2 (8.0–8.3)	—	—	—	—
**5-question disability^a^**	120,565	22.7 (22.4–22.9)	—	—	—	—
Ambulatory disability	82,978	13.8 (13.6–14.0)	—	—	—	—
Cognitive disability	48,695	10.6 (10.4–10.8)	—	—	—	—
Vision disability	24,766	5.0 (4.8–5.1)	—	—	—	—
Self-care disability	20,665	3.8 (3.7–3.9)	—	—	—	—
Independent living disability	36,971	6.7 (6.6–6.9)	—	—	—	—

Abbreviations: —, does not apply; CI, confidence interval.

a Disability categories are not mutually exclusive.

b Two or more of the health conditions presented here: arthritis, heart disease, diabetes, obesity, depression, hypertension.

c Smoked at least 100 cigarettes in their lifetime and currently smoked either every day or some days.

d Consumed at least 5 drinks (men) or at least 4 drinks (women) on an occasion one or more times during the past 30 days.

e Participated in ≥150 minutes of moderate-intensity equivalent aerobic activity per week.

There were differences in the prevalence of many respondent characteristics between the 2 disability measures that were not mutually exclusive. Notably, compared with adults whose disability was identified by the 2-question measure, the group whose disability was identified by the 5-question measure had a higher proportion of people aged 18 to 24 (8.5% vs 5.7%) and Hispanic adults (16.8% vs 11.4%). Although a lower prevalence of arthritis (51.8% vs 56.6%) was seen among people whose disability was identified by the 5-question set compared with people whose disability was identified by the 2-question set, the prevalence of heart disease, diabetes, obesity, and fair or poor self-rated health was similar for the 2 groups (Table 1).

Among adults reporting disability, about half (51.2%) reported having disability with both the 2-question and the 5-question set, while 22.6% reported disability only in response to the 2-question set and 26.3% reported disability only in response to the 5-question set (*r *= 0.60; *P* < .001; data not shown). Adults with a disability according to both measures had a higher prevalence of health conditions and poor health status indicators compared with people whose disability was identified by only one of the measures. For example, nearly two-thirds (62.1%) of adults identified as having a disability using both measures had arthritis compared with 44.3% (95% CI, 43.3%–45.3%) of people whose disability was identified only by the 2-question set and 31.8% (95% CI, 30.8%–32.8%) of people whose disability was identified only by the 5-question set (Table 2). However, the pattern of health behaviors was less consistent. For example, the highest prevalence of smoking was seen among people identified with a disability by both measures (28.5% vs 18.0% for the 2-question only set and 25.1% for the 5-question only set) and the highest prevalence of binge drinking was seen among people whose disability was identified only by the 5-question set (16.9% vs 10.2% for both sets and 14.8% for the 2-question only set) (Table 2).

**Table 2:** Prevalence of Selected Demographic and Health Characteristics, by Mutually Exclusive Disability Category Among Adults Aged 18 Years or Older, United States and Territories, 2013 Behavioral Risk Factor Surveillance System

Characteristic	Both 2-Question and 5-Question Disability (N = 86,779)		2-Question Disability Only (N = 37,771)		5-Question Disability Only (N = 33,786)	
	n	Weighted % (95% CI)	n	Weighted % (95% CI)	n	Weighted % (95% CI)
**Age group, y**						
18–24	1,330	4.8 (4.4–5.3)	1,117	7.5 (6.9–8.2)	2,218	15.7 (14.7–16.6)
25–44	9,581	20.3 (19.6–20.9)	5,536	24.3 (23.3–25.3)	6,037	29.4 (28.4–30.5)
45–64	36,501	44.1 (43.4–44.8)	15,404	40.2 (39.2–41.2)	10,823	29.9 (28.9–31.0)
≥65	39,367	30.8 (30.2–31.4)	15,714	28.0 (27.1–28.9)	14,708	25.0 (24.2–25.8)
**Sex**						
Male	30,490	43.0 (42.3–43.7)	17,593	52.8 (51.7–53.8)	11,649	41.8 (40.7–42.9)
Female	56,289	57.0 (56.3–57.7)	20,178	47.2 (46.2–48.3)	22,137	58.2 (57.1–59.4)
**Race/ethnicity**						
White, non-Hispanic	65,734	67.5 (66.7–68.2)	31,742	76.4 (75.2–77.5)	22,574	52.7 (51.5–53.9)
Black, non-Hispanic	8,499	14.2 (13.7–14.8)	2,091	8.6 (7.9–9.2)	3,736	14.8 (14.0–15.7)
Hispanic	5,563	12.5 (11.9–13.1)	1,484	9.1 (8.3–10.0)	4,541	25.3 (24.2–26.5)
Other, non-Hispanic	5,466	5.8 (5.4–6.3)	1,851	6.0 (5.3–6.9)	2,381	7.2 (6.5–8.0)
**Health conditions**						
Arthritis	58,607	62.1 (61.3–62.8)	19,153	44.3 (43.3–45.3)	14,464	31.8 (30.8–32.8)
Heart disease	18,829	19.5 (19.0–20.1)	4,717	10.0 (9.5–10.6)	4,103	9.3 (8.8–9.9)
Diabetes	23,161	24.7 (24.1–25.3)	5,427	13.7 (12.9–14.6)	6,116	14.8 (14.0–15.6)
Obesity	35,748	43.2 (42.5–44.0)	12,013	33.7 (32.7–34.7)	10,974	34.3 (33.2–35.4)
Depression	37,466	46.5 (45.8–47.2)	8,745	22.7 (21.8–23.5)	9,809	30.1 (29.1–31.1)
Hypertension	54,056	57.0 (56.3–57.7)	18,257	43.0 (41.9–44.0)	16,990	40.8 (39.7–41.9)
≥2 Chronic conditions^a^	67,534	74.3 (73.6–74.9)	21,129	50.2 (49.1–51.3)	19,129	47.3 (46.2–48.4)
**Health behaviors**						
Current smoking^b^	20,508	28.5 (27.8–29.1)	5,564	18.0 (17.2–18.9)	7,159	25.1 (24.2–26.1)
Binge drinking^c^	5,962	10.2 (9.7–10.6)	4,121	14.8 (14.0–15.6)	3,916	16.9 (16.0–17.8)
Flu vaccine	43,094	46.7 (45.9–47.4)	19,092	46.0 (45.0–47.1)	14,283	37.1 (36.0–38.3)
Sufficient aerobic physical activity^d^	26,267	33.1 (32.4–33.8)	18,657	50.8 (49.7–51.9)	13,391	42.9 (41.8–44.1)
**Health status**						
Fair or poor self-rated health	49,609	57.6 (56.9–58.3)	8,408	23.2 (22.3–24.1)	9,686	29.3 (28.3–30.3)
≥14 Poor physical health days	39,362	47.8 (47.0–48.5)	6,326	17.7 (16.9–18.5)	4,781	14.4 (13.6–15.2)
≥14 Poor mental health days	23,697	33.5 (32.8–34.2)	3,532	11.1 (10.4–11.8)	5,723	21.4 (20.4–22.4)

Abbreviation: CI, confidence interval.

a Two or more of the health conditions presented here: arthritis, heart disease, diabetes, obesity, depression, hypertension.

b Smoked at least 100 cigarettes in their lifetime and currently smoked either every day or some days.

c Consumed at least 5 drinks (men) or at least 4 drinks (women) on an occasion one or more times during the past 30 days.

d Participated in ≥150 minutes of moderate-intensity equivalent aerobic activity per week.

Adults with disability identified only by the 2-question set or only by the 5-question set also differ by many demographic and health characteristics. Compared with adults whose disability was identified only by the 2-question set, the group whose disability was identified only by the 5-question set had a higher proportion of adults aged 18 to 24 (15.7% vs 7.5%), female adults (58.2% vs 47.2%), and Hispanic adults (25.3% vs 9.1%). They also had a higher prevalence of depression (30.1% vs 22.7%), current smoking (25.1% vs 18.0%), and having 14 or more poor mental health days (21.4% vs 11.1%). Adults whose disability was identified only by the 5-question set had a lower prevalence of arthritis (31.8% vs 44.3%), receipt of a flu vaccine in the past year (37.1% vs 46.0%), and engaging in sufficient aerobic physical activity (42.9% vs 50.8%) compared with adults identified only by the 2-question set (Table 2).

When comparing the 5 mutually exclusive disability types, adults with only ambulatory disability had the highest proportion of adults aged 65 or older (45.3%) and the highest prevalence of chronic conditions such as arthritis (63.6%) and hypertension (61.5%). Adults with only cognitive disability had the highest proportion of adults aged 18 to 24 years (23.3%) and the highest prevalence of depression (50.0%), current smoking (31.1%), binge drinking (21.7%), and 14 or more poor mental health days (35.2%). More than one-quarter (26.7%) of adults with only vision disability were Hispanic, which was higher than the proportion of Hispanics among other disability types. Adults with only self-care disability had the highest proportion of male adults (56.1%) compared with other disability types. Adults with 2 or more disability types had the highest prevalence of depression and fair or poor self-rated health (Table 3).

**Table 3:** Prevalence of Selected Demographic and Health Characteristics, by Mutually Exclusive 5-Question Disability Types Among Adults Aged 18 Years or Older, United States and Territories, 2013 Behavioral Risk Factor Surveillance System

Characteristic	Ambulatory Disability Only (n = 36,350)	Cognitive Disability Only (n = 17,711)	Vision Disability Only (n= 7,535)	Self-Care Disability Only (n = 1,073)	Independent Living Disability Only (n = 3,164)	≥2 Disability Types (n = 54,732)
	Weighted % (95% Confidence Interval)					
**Age group, y**						
18–24	2.0 (1.6–2.4)	23.3 (21.9–24.8)	12.9 (11.0–15.0)	7.5 (4.2–13.0)	12.3 (9.9–15.2)	4.8 (4.3–5.3)
25–44	13.3 (12.4–14.2)	38.1 (36.6–39.5)	27.5 (25.3–29.9)	24.4 (18.2–31.7)	35.4 (31.7–39.3)	21.2 (20.4–22.0)
45–64	39.5 (38.4–40.6)	25.4 (24.2–26.6)	37.1 (34.7–39.4)	45.2 (37.9–52.7)	27.1 (23.7–30.8)	46.4 (45.5–47.3)
≥65	45.3 (44.2–46.4)	13.3 (12.6–14.1)	22.6 (20.9–24.4)	22.9 (18.5–28.0)	25.2 (22.6–28.0)	27.7 (27.0–28.4)
**Sex**						
Male	41.6 (40.5–42.7)	46.4 (44.9–47.9)	48.7 (46.2–51.1)	56.1 (48.9–63.1)	37.1 (33.3–41.0)	40.5 (39.6–41.4)
Female	58.4 (57.3–59.5)	53.6 (52.1–55.1)	51.3 (48.9–53.8)	43.9 (36.9–51.1)	62.9 (59.0–66.7)	59.5 (58.7–60.4)
**Race/ethnicity**						
White, non-Hispanic	68.8 (67.6–70.0)	62.2 (60.6–63.8)	49.5 (47.0–52.0)	59.4 (51.2–67.1)	58.8 (54.7–62.8)	61.5 (60.5–62.4)
Black, non-Hispanic	13.8 (13.0–14.7)	11.3 (10.2–12.4)	14.7 (12.9–16.7)	15.0 (8.9–24.1)	11.6 (9.2–14.4)	16.3 (15.6–17.1)
Hispanic	12.5 (11.5–13.6)	19.0 (17.6–20.5)	26.7 (24.4–29.1)	19.2 (13.2–27.1)	22.7 (18.9–27.1)	16.2 (15.4–17.0)
Other, non-Hispanic	4.9 (4.4–5.5)	7.5 (6.6–8.5)	9.1 (7.3–11.3)	6.5 (3.9–10.5)	6.9 (5.1–9.2)	6.0 (5.5–6.6)
**Health conditions**						
Arthritis	63.6 (62.4–64.7)	26.2 (25.0–27.5)	24.9 (23.0–26.9)	49.1 (41.7–56.5)	32.5 (29.1–36.1)	62.3 (61.4–63.2)
Heart disease	18.5 (17.7–19.3)	6.7 (6.1–7.4)	9.0 (7.8–10.3)	6.7 (4.6–9.6)	11.5 (9.5–13.9)	20.6 (19.9–21.3)
Diabetes	25.3 (24.3–26.3)	9.3 (8.5–10.2)	12.7 (11.3–14.2)	17.1 (11.2–25.2)	12.1 (10.1–14.5)	26.5 (25.8–27.3)
Obesity	48.6 (47.4–49.7)	28.5 (27.2–29.8)	29.5 (27.3–31.8)	34.9 (27.2–43.3)	26.7 (23.2–30.5)	43.5 (42.6–44.4)
Depression	21.9 (21.1–22.8)	50.0 (48.5–51.5)	17.7 (15.9–19.7)	24.1 (18.8–30.4)	34.3 (30.9–37.8)	52.8 (51.9–53.6)
Hypertension	61.5 (60.4–62.6)	30.9 (29.6–32.2)	38.7 (36.4–41.1)	45.4 (38.4–52.7)	39.2 (35.8–42.8)	58.0 (57.1–58.9)
≥2 Chronic conditions^a^	73.1 (72.0–74.0)	43.6 (42.2–45.1)	38.1 (35.9–40.4)	55.3 (48.0–62.3)	45.5 (41.8–49.3)	76.3 (75.5–77.1)
**Health behaviors**						
Current smoking^b^	19.3 (18.5–20.2)	31.1 (29.7–32.5)	23.3 (21.3–25.5)	18.6 (14.5–23.5)	25.7 (22.6–29.0)	31.3 (30.5–32.1)
Binge drinking^c^	8.9 (8.2–9.6)	21.7 (20.4–23.0)	15.4 (13.6–17.3)	14.3 (8.5–23.0)	13.4 (11.0–16.3)	9.8 (9.2–10.4)
Flu vaccine	51.9 (50.7–53.0)	34.3 (32.9–35.8)	33.3 (31.1–35.7)	40.2 (33.1–47.8)	39.7 (36.1–43.5)	44.6 (43.7–45.5)
Sufficient aerobic physical activity^d^	36.7 (35.6–37.8)	47.7 (46.1–49.3)	46.6 (44.0–49.2)	47.3 (39.7–55.1)	40.4 (36.6–44.3)	28.9 (28.1–29.8)
**Health status**						
Fair or poor self-rated health	42.5 (41.4–43.5)	27.7 (26.4–29.1)	23.8 (21.8–25.9)	31.9 (26.0–38.3)	36.1 (32.5–39.9)	65.5 (64.6–66.4)
≥14 Poor physical health days	30.2 (29.2–31.2)	15.6 (14.6–16.7)	11.2 (9.9–12.6)	31.2 (24.8–38.5)	27.7 (24.6–31.2)	54.4 (53.5–55.3)
≥14 Poor mental health days	12.5 (11.7–13.3)	35.2 (33.7–36.7)	13.2 (11.4–15.3)	22.0 (14.6–31.7)	23.7 (20.3–27.4)	40.2 (39.3–41.1)

a Two or more of the health conditions presented here: arthritis, heart disease, diabetes, obesity, depression, hypertension.

b Smoked at least 100 cigarettes in their lifetime and currently smoked either every day or some days.

c Consumed at least 5 drinks (men) or at least 4 drinks (women) on an occasion one or more times during the past 30 days.

d Participated in ≥150 minutes of moderate-intensity equivalent aerobic activity per week.

## Discussion

Overall disability prevalence estimates using the 2 disability measures were similar. However, there was an approximate 50% overlap in adults identifying as having a disability using both measures. Although adults who reported disability with each of the question sets (2-question measure and 5-question measure) had many similar characteristics, several differences in demographic and health characteristics were seen in adults who identified as having a disability by one of the question sets and not the other.

Previous reports and studies have compared disability prevalence estimates; however, most involved comparisons of different sample populations (13–15), survey years (14–16), modes of data collection (14,16), or used small sample sizes (19,20). Our study provides a unique contribution to the literature as the only difference was in the measure of disability — the sample population, survey year, and mode of survey administration were the same. Thus, any differences we found probably resulted from the questions used to assess disability. As in our study, previous studies found that using different operational measures of disability resulted in different disability prevalence estimates. Although in some cases the differences in the estimates were small, particularly when the question sets attempted to measure the same concepts, the populations identified through the different question sets were not the same groups of individuals (15,21).

We found that a high proportion of those who identified as having a disability using the 2-question set were older adults and more than half reported arthritis. Similarly, nearly half of adults with an ambulatory disability were older adults (≥65 years) and nearly 2 of 3 reported having arthritis. The similarity in characteristics reported by those with 2-question disability and those with an ambulatory disability is not surprising in light of a recent study that identified the most common conditions reported by respondents with 2-question disability as musculoskeletal conditions such as arthritis and back problems and the most common limitation as difficulty walking (22). This study also found that these same respondents did not commonly report cognitive and vision limitations as a cause of their disability (22).

We found that the 5-question disability measure captured a higher proportion of younger adults, Hispanics, and those reporting poor mental health (depression and ≥14 poor mental health days). Some of these differences can likely be attributed to characteristics of respondents who identified as having a cognitive disability. This group had the highest proportion of younger adults; nearly one-quarter of those with a cognitive disability were aged 18 to 24. In comparison, only 2.0% of those with an ambulatory disability and 12.9% of those with a vision disability were aged 18 to 24. Prior cognitive testing of this question revealed that although it captured a range of conditions, mental illness was often cited as a reason for reporting difficulty in this domain (23). Therefore, it is not unexpected that in our study those with cognitive disability reported the highest prevalence of depression and 14 or more poor mental health days compared with the other disability types. These respondents also reported the highest prevalence of current smoking and binge drinking, which is consistent with findings of studies showing a higher prevalence of cigarette smoking and binge drinking among those with mental illness compared with those without (22,24).

Somewhat unexpectedly, we found a higher proportion of Hispanic adults among those with vision disability than the other disability types. On further exploration and review of both English and Spanish versions of the foundational questionnaire, we discovered a difference in the question about vision disability. The English version of the question asked about “serious” difficulty seeing (“Are you blind or do you have serious difficulty seeing, even when wearing glasses?”), whereas the Spanish version translated to “some” difficulty seeing (“¿Es ciego o tiene alguna dificultad para ver, aun cuando usa lentes?”) (Spanish translation has been corrected from 2015 onward.). States are able to make changes to the foundational Spanish questionnaire to reflect the prevalent dialect of their population; however, this discrepancy between the English and Spanish translations of the vision disability question may account for much of the higher prevalence of Hispanic ethnicity we found among those with a vision disability. In a sensitivity analysis where we excluded respondents who completed the Spanish questionnaire, we found some expected differences (eg, a lower proportion of Hispanic respondents among all disability categories) when we compared these analyses to analyses completed using the full data set. We also found some differences in estimates of health behaviors and health status for some of the specific disability types. However, most of these differences were small (ie, less than 2 percentage points different from the original estimate) and nonsignificant on the basis of overlapping 95% confidence intervals. States, in particular those with large Spanish-speaking populations, should interpret results for this question with caution.

This study has 4 primary limitations. First, the data were self-reported and are subject to recall and social desirability bias. Nonetheless, self-reported data from surveillance systems are routinely used to assess disability at the national and state level. Second, we do not have information on the permanence, duration, or underlying medical condition of the disability and cannot assess to what extent, if any, this information might explain some of the prevalence differences noted in this study. Third, BRFSS does not include adults living in institutional settings or group homes or allow for proxy respondents, which might systematically exclude people with disabilities. This may result in an underestimation of disability prevalence. Fourth, 2013 BRFSS does not include the sixth question in the HHS-recommended disability set, “Are you deaf or do you have serious difficulty hearing?” largely because the survey is administered by the telephone. Inclusion of this question likely would increase the prevalence of disability if added to the 5-question measure, as prevalence estimates of hearing disability obtained from other surveys using all 6 questions range from 1.9% to 3.9%, depending on the data set and study population (15,25–27). This will be examined further once 2016 BRFSS data are made available.

Using public health surveillance data to identify individuals living with disability is a critical step in understanding and addressing the health needs and reducing the health disparities experienced by this population. Because disability is a complex, multidimensional concept that may not be fully captured in a few questions on a survey, researchers and public health practitioners need to be explicit in stating how disability is operationalized in their work. States using BRFSS data to identify adults with disabilities may wish to conduct similar analyses to understand the implications of reporting on the basis of one disability measure versus another. The finding of similar prevalence of health characteristics by different disability measures suggests a consistency in patterns of key health disparities experienced by adults with disabilities and points to the importance of intervention through public health policy and practice to address these disparities. Although the overall disability estimates from the 2 measures in our study are similar, the 5-question set allows for identification of health needs and behaviors associated with certain disability types (eg, ambulatory, cognitive, vision). This level of detail can help develop targeted interventions and programs and guide the adaptation of existing health promotion programs to be more inclusive of adults who experience specific types of disabilities.
